# Willingness to adopt gene therapy for Duchenne muscular dystrophy: barriers, facilitators, and multidisciplinary readiness

**DOI:** 10.3389/fmed.2026.1985172

**Published:** 2026-10-21

**Authors:** Rocío García-Uzquiano, María Branas-Pampillón, Julita Medina-Cantillo, Beatriz García-Palop, Elena Ruiz-Beato, Jorge Maurino, José Luis Poveda-Andrés

**Affiliations:** 1 Department of Pediatric Neurology, Hospital General Universitario Gregorio Marañón, Madrid, Spain; 2 Medical Department, Roche Farma, Madrid, Spain; 3 Department of Rehabilitation, Hospital Sant Joan de Déu, Barcelona, Spain; 4 Department of Pharmacy, Hospital Universitari Vall D'Hebron, Barcelona, Spain; 5 Health Economics and Outcome Research Unit, Roche Farma, Madrid, Spain; 6 Management Department, Hospital Universitari i Politècnic La Fe, Valencia, Spain

**Keywords:** duchenne muscular dystrophy, Gene Therapy, health-system readiness, interprofessional decision-making, public health policy, Six-minute walk test

## Abstract

**Introduction:**

Management of Duchenne muscular dystrophy (DMD) is shifting toward genotype-specific, disease-modifying interventions, including adeno-associated virus-mediated micro-dystrophin gene transfer. Grounded in established technology adoption frameworks, this study evaluated healthcare professionals' (HCPs) willingness to adopt advanced gene therapies, identified critical systemic barriers and facilitators, and mapped multidisciplinary familiarity and quality perceptions regarding functional outcome measures.

**Methods:**

We conducted a cross-sectional, web-based study evaluating 53 HCPs actively involved in DMD care across Spain: 37 pediatric neurologists, 11 rehabilitation physicians, and 5 hospital pharmacists. Using ad-hoc questionnaires and validated psychometric instruments, we evaluated technical knowledge, functional scale perceptions (including sensitivity to change and specificity), behavioral intent, and implementation challenges. Multivariate logistic regression identified independent predictors of high adoption willingness.

**Results:**

Receptivity was high, with 67.6% of pediatric neurologists demonstrating strong willingness to adopt gene therapy. Clinicians ranked acute post-infusion safety concerns (mean rank: 2.30; lower values indicate greater relevance) and uncertainty regarding long-term efficacy (mean rank: 2.70) as primary clinical barriers, followed by institutional cost constraints (mean rank: 3.38). Conversely, the availability of robust clinical trial evidence was the primary operational facilitator (86.5%). In the multivariate model, a clinician′s perception of the Six-Minute Walk Test as an objective and sensitive instrument emerged as the sole independent predictor of high adoption intent (OR = 15.23, 95%CI: 1.58-146.67, *p* = 0.018).

**Conclusions:**

HCPs demonstrate high theoretical receptivity to DMD gene therapy. However, clinical translation remains constrained by operational bottlenecks, acute safety risks, and technical gaps in functional outcome mastery. Because clinician readiness depends on diagnostic confidence in outcome measures, health authorities must prioritize standardized safety surveillance protocols, interprofessional training on scale psychometrics, and transparent reimbursement frameworks prior to broader deployment.

## 1 Introduction

Duchenne muscular dystrophy (DMD) is a fatal, X-linked recessive neuromuscular disorder characterized by progressive muscle degeneration and premature mortality (1, 2). As the most prevalent muscular dystrophy in childhood, affecting approximately 7.1 per 100,000 live male births, it imposes a profound clinical and economic burden on public health systems (1, 3, 4). Symptoms typically manifest between 2 and 3 years of age as delayed motor milestones and proximal muscle weakness (1, 5–7). The disease predictably advances to a loss of independent ambulation and multisystem deterioration, culminating in fatal respiratory or cardiac complications between the second and fourth decades of life. Standard multidisciplinary management and optimized corticosteroid regimens have altered this trajectory, extending independent ambulation from a historical mean of 8.5 years to approximately 11–14 years (1, 3, 8). Recently, disease-modifying strategies targeting the underlying genetic pathology have expanded the therapeutic landscape, although they introduce unprecedented clinical, structural, and economical complexities to healthcare networks (1, 9–11).

From a public health policy perspective, these gene therapies present implementation challenges that extend beyond traditional clinical efficacy metrics (12). Structurally, broader-scale deployment across specialized referral centers demands advanced institutional infrastructure, including secure viral vector cold-chain storage, dedicated infusion units, intensive acute-care monitoring capacity, and highly coordinated interprofessional teams (12, 13). Serious safety considerations further complicate integration into public hospital networks; immune-mediated adverse events occur in approximately 30% of treated patients, including severe hepatotoxicity, myocarditis, thrombotic microangiopathy, and treatment-related mortality (14). Consequently, regulatory frameworks mandate intensive long-term safety surveillance spanning 5–15 years to track delayed toxicities and immunogenic responses (15). Additionally, the high cost of these therapies imposes unprecedented budget impacts on healthcare systems (16). Sustainable deployment therefore relies on health-system resilience and the operational alignment of multidisciplinary stakeholders, including hospital pharmacists who manage logistical coordination and local health technology assessments (17–19).

Currently, delandistrogene moxeparvovec is the sole gene therapy available for DMD (20–23). This recombinant adeno-associated virus vector delivers a functional micro-dystrophin gene assembly directly to target muscle tissues. However, following an unfavorable opinion from the European Medicines Agency (EMA), this therapeutic lacks regulatory approval in Europe, restricting access strictly to clinical trial settings (24). This regulatory latency creates a unique, highly informative window for health authorities and decision-makers.

Grounded in established technology adoption frameworks, including the Technology Acceptance Model (TAM), the Unified Theory of Acceptance and Use of Technology (UTAUT), and models of healthcare innovation diffusion, a clinician's intention to adopt a medical innovation is governed by perceived usefulness, effort expectancy, and user-specific readiness (25–27). Although theoretical receptivity toward genotype-guided advanced therapies is high, the lack of real-world exposure highlights the need to capture healthcare professionals' (HCPs) perspectives on the anticipated challenges of integrating gene therapy into routine DMD care (28). Driven by this public health priority, this study evaluated pediatric neurologists' willingness to adopt advanced gene therapies, identified critical systemic barriers and facilitators, and determined whether professional characteristics influence clinical readiness.

## 2 Methods

### 2.1 Study design

This non-interventional, cross-sectional, web-based study was conducted from July to December 2025 to evaluate HCPs involved in the management of DMD. Eligible participants included pediatric neurologists and rehabilitation physicians actively managing patients with DMD in a hospital setting. Hospital pharmacists were eligible if they possessed direct experience with advanced therapies or specific DMD treatments. In collaboration with the Spanish Society of Pediatric Neurology (SENEP), the Spanish Society of Child Rehabilitation (SERI), and the Catalan Society of Clinical Pharmacy (SCFC), participants were invited via email. The Institutional Review Board of Hospital Clínico San Carlos (Madrid, Spain), approved the study protocol, which adhered strictly to the Declaration of Helsinki. All participants provided electronic informed consent.

### 2.2 Outcome measures and questionnaires

Data were collected using a secure online electronic data capture platform. The survey comprised ad-hoc questionnaires and validated psychometric instruments, structured into three distinct domains. First, functional tool perceptions were evaluated across all participants to assess baseline capacity for fine-grained outcome tracking, which is essential under post-marketing surveillance and value-based reimbursement frameworks to verify real-world efficacy. Second, willingness, barriers, and facilitators regarding gene therapy implementation were measured to map operational bottlenecks. Third, work-related psychosocial and behavioral measures were evaluated to identify predictors of adoption (29, 30); these were restricted to pediatric neurologists as the primary prescribers initiating disease-modifying treatment.

**Knowledge, utilization, and quality perception of evaluation tools:** This section evaluated seven functional scales: the North Star Ambulatory Assessment (NSAA), Six-Minute Walk Test (6MWT), Four-Stair Climb/Down (4SC/4SD), Performance of Upper Limb (PUL), Ten-Meter Walk-Run Test, Time to Rise from Floor (TTRF), and Egen Klassifikation 2 (EK-2) (3, 31). Respondents assessed familiarity, routine clinical and trial utilization, and specific quality attributes: relevance, quantifiability, feasibility, validity, objectivity, sensitivity to change (ability to detect clinically meaningful functional change over time), and specificity (ability to isolate disease-specific motor deficits without performance confounding).

**Gene therapy willingness, barriers, and facilitators:** This section examined baseline deployment of gene therapies in neuromuscular diseases alongside perceived usefulness, ease of use, attitude towards use, and behavioral intention regarding the adoption of gene therapy to treat DMD. Operational facilitators and structural barriers to adoption were mapped via ranked, multi-response item arrays. The specific categories included in these arrays were derived from a synthesis of published literature on gene and advanced therapy implementation across other genetic and neuromuscular disorders, combined with the multidisciplinary expertise of the research team across pediatric neurology, hospital pharmacy, and rehabilitation medicine. Candidate items were iteratively refined to ensure clinical relevance, comprehensiveness, and contextual alignment with national hospital infrastructure.

**Psychosocial and work-related measures:** To evaluate psychological profiles, professional attitudes, and well-being, pediatric neurologists completed the following validated instruments: the Evidence-Based Practice Attitudes Scale (EBPAS), the Regret Intensity Scale (RIS-10), the Jefferson Scale of Physician Empathy-Health Practitioners (JSPE-HP), the Utrecht Work Engagement Scale (UWES), and a single-item measure of occupational burnout (32–36). Receptiveness toward the adoption of evidence-based innovations was evaluated using the 15-item EBPAS (32). This tool assesses four primary dimensions (Openness, Requirements, Divergence, and Appeal) using a 5-point Likert scale ranging from 0 (“not at all”) to 4 (“completely agree”). Scores yield a global range of 0 to 4, where higher values indicate a greater willingness to adopt innovations. The RIS-10 evaluated the psychological and emotional sequelae of a major clinical regret over the preceding 5 years (33). Participants rated their current feelings across 10 items using a 5-point Likert scale (1, strongly disagree; 5, strongly agree). Total score ranges from 10 to 50, with scores exceeding 30 classifies as moderate-to-high regret intensity. Patient-oriented empathy was measured using the 20-item JSE-HP (34). Responses were gathered on a 7-point Likert scale (1, totally disagree; 7, totally agree), yielding total scores from 20 to 140. Higher values reflect greater empathic engagement. Work engagement, defined as a positive, fulfilling, work-related state of mind characterized by vigor, dedication, and absorption, was assessed using the 9-item short form of the UWES (35). Items were scored on a 7-point operational frequency scale ranging from 0 to 6. Professional burnout was measured using a single-item instrument from the Physician Work Life Study (36). This 5-point ordinal scale ranges from 1 (“I enjoy my work; I have no symptoms of burnout”) to 5 (“I feel completely burned out and often wonder if I can go on”). Scores ≥3 indicate emotional exhaustion and serve as the threshold for manifest burnout.

### 2.3 Statistical analysis

Continuous variables, including respondent age, patient volumes, annual consultation frequencies, and years of professional experience, were evaluated for normality. Normally distributed data are expressed as means with standard deviations (SD), whereas skewed distributions are summarized as medians with interquartile ranges (IQR), with absolute minimum and maximum values. These continuous reporting standards were also applied to global and subscale scores derived from psychometric instruments. Categorical variables are presented as absolute and relative frequencies (percentages). Evaluated parameters encompassed institutional practice settings, clinical infrastructure availability, history of research engagement, and discrete quality-attribute scale appraisals formatted as “Yes”, “No”, or “No opinion”. Validated clinical thresholds categorized respondents into binary or ordinal strata for psychometric screening, including regret intensity and categorical empathy, work engagement, or burnout.

Descriptive data and behavioral outcomes were cross-tabulated and stratified by independent factors: clinician experience levels bifurcated by sample medians, patient load volumes, and the utilization of dedicated neuromuscular consultations. To identify independent predictors of a high willingness to adopt or recommend gene therapies, we constructed a multivariate logistic regression model. Covariates included participants′ demographic and professional characteristics, stakeholder type, patient volume, and technical scale confidence metrics. Effect sizes are reported as odds ratios (OR) with corresponding 95% confidence intervals (CI). All statistical tests were two-sided, and the significance threshold was established *a priori* at alpha = 0.05.

## 3 Results

A total of 53 HCPs completed the study: 37 pediatric neurologists (69.8%), 11 rehabilitation physicians (20.8%), and 5 hospital pharmacists (9.4%). Clinical practice was predominantly centered in tertiary referral hospitals across all three cohorts. Dedicated neuromuscular consultations were utilized by approximately half of the practicing clinicians. Research engagement was low across the sample, with fewer than 20% of respondents participating in clinical trials. Detailed sociodemographic and professional characteristics are provided in Table 1.

**Table 1:** Sociodemographic and professional characteristics of the sample (*n* = 53).

Characteristics	Pediatric neurologists (*n* = 37)	Rehabilitation physicians (*n* = 11)	Hospital pharmacists (*n* = 5)
Age, years, mean (SD)	42.5 (7.0)	50.2 (7.4)	46.6 (7.4)
Female, n (%)	25 (67.6)	10 (90.9)	4 (80.0)
Years of professional experience, mean (SD)	13.7 (6.9)	20.3 (8.0)	19.4 (6.9)
DMD clinical experience, years, mean (SD)	11.8 (6.2)	15.4 (7.5)	9.8 (9.2)
Type of hospital, tertiary, n (%)	20 (54.1)	7 (63.6)	4 (80.0)
Full dedication to neuromuscular care, n (%)	17 (45.9)	5 (45.5)	NA
DMD patient volume, median (IQR)	4.0 (2.0-16.0)	10.0 (5.0-20.0)	NA
Frequency of follow-up visits, month, median (IQR)	6.0 (4.0-6.0)	6.0 (4.0-6.0)	NA
DMD trial investigator, n (%)	7 (18.9)	2 (18.2)	NA
DMD publication authorship (past 3 years), n (%)	8 (21.6)	0	1 (20.0)

DMD, Duchenne Muscular Dystrophy; SD, standard deviation.

The behavioral profiles of the participating pediatric neurologists reflected high professional openness to evidence-based innovations (mean global EBPAS score: 1.8) and strong occupational engagement (mean global UWES score: 4.5) (Table 2). Conversely, professional burnout was reported by 27.0% of participants.

**Table 2:** Behavioral and psychosocial characteristics of pediatrics neurologists (*n* = 37).

Variable	Score
EBPAS score, mean (SD)	1.8 (0.3)
JES-HP score, mean (SD)	121.9 (14.6)
RIS-10 score, mean (SD)	20.3 (7.6)
UWES score, mean (SD)	4.5 (0.9)
Professional burnout, n (%)	10 (27.0)

EBPAS, Evidence-Based Practice Attitudes Scale; JSE-HO, Jefferson Empathy Scale; UWES, Utrecht Work Engagement Scale; RIS-10, Regret Intensity Scale.

### 3.1 Familiarity, clinical utilization, and perceived quality of functional scales

Core motor function tools achieved near-universal familiarity, with over 90% of clinicians recognizing the NSAA and the 6MWT (Table 3). Awareness ranged from 78.4% to 83.8% among pediatric neurologists for secondary functional tools, including the 4SC/4SD, PUL, 10MWRT, and TTRF. Hospital pharmacists exhibited lower familiarity with these specialized tools (awareness: 60%-80%), except for the 6MWT (100% familiarity).

**Table 3:** Familiarity, clinical utilization, and quality perception of functional motor scales in DMD.

Scale	Discipline	Familiarity n (%)	Utilization in trials n (%)	Utilization in practice n (%)	Perceived relevance n (%)	Perceived validity n (%)	Perceived objectivity n (%)
NSAA	Pediatric neurologist	35 (94.6)	20 (57.1)	32 (91.4)	35 (100.0)	33 (94.3)	31 (88.6)
	Rehabilitation physician	11 (100.0)	5 (45.5)	10 (90.9)	11 (100.0)	10 (90.9)	9 (81.8)
	Hospital pharmacist	4 (80.0)	3 (75.0)	3 (75.0)	4 (100.0)	3 (75.0)	3 (75.0)
6MWT	Pediatric neurologist	36 (97.3)	18 (50.0)	33 (91.7)	35 (97.2)	32 (88.9)	28 (77.8)
	Rehabilitation physician	11 (100.0)	6 (54.5)	10 (90.9)	10 (90.9)	10 (90.9)	9 (81.8)
	Hospital pharmacist	5 (100.0)	3 (60.0)	4 (80.0)	5 (100.0)	4 (80.0)	4 (80.0)
4SC/4SD	Pediatric neurologist	30 (81.1)	15 (50.0)	21 (70.0)	27 (90.0)	25 (83.3)	22 (73.3)
	Rehabilitation physician	10 (90.9)	6 (60.0)	9 (90.0)	9 (90.0)	9 (90.0)	8 (80.0)
	Hospital pharmacist	3 (75.0)	3 (60.0)	3 (75.0)	2 (50.0)	3 (75.0)	2 (66.7)
PUL	Pediatric neurologist	31 (83.8)	14 (45.2)	18 (58.1)	26 (83.9)	24 (77.4)	21 (67.7)
	Rehabilitation physician	11 (100.0)	4 (36.4)	6 (54.5)	9 (81.8)	8 (72.7)	7 (63.6)
	Hospital pharmacist	3 (75.0)	4 (80.0)	1 (25.0)	1 (25.0)	3 (75.0)	3 (100.0)
EK-2	Pediatric neurologist	23 (62.2)	11 (47.8)	13 (56.5)	15 (67.6)	13 (59.5)	11 (48.6)
	Rehabilitation physician	11 (100.0)	5 (45.5)	7 (63.6)	8 (72,7)	7 (63.6)	6 (54.5)
	Hospital pharmacist	3 (60.0)	2 (66.7)	1 (33.3)	3 (60.0)	2 (40.0)	2 (40.0)
10MWRT	Pediatric neurologist	31 (83.8)	12 (38.7)	16 (51.6)	26 (70.3)	25 (67.6)	25 (67.6)
	Rehabilitation physician	10 (90.9)	6 (60.0)	9 (90.0)	8 (72.7)	7 (63.6)	8 (72.7)
	Hospital pharmacist	4 (80.0)	3 (75.0)	3 (75.0)	3 (60.0)	3 (60.0)	0 (0.0)
TTRF	Pediatric neurologist	29 (78.4)	15 (51.7)	24 (82.8)	31 (83.8)	29 (78.4)	26 (70.3)
	Rehabilitation physician	11 (100.0)	6 (54.5)	9 (81.8)	10 (90.9)	10 (90.9)	9 (81.8)
	Hospital pharmacist	3 (60.0)	2 (66.7)	3 (100.0)	4 (80.0)	2 (40.0)	1 (20.0)

DMD, Duchenne Muscular Dystrophy; EK-2, Egen Klassifikation 2; NSAA, North Star Ambulatory Assessment; PUL, Performance of Upper Limb; TTRF, Time to Rise from Floor; 4SC, Four-Stair Climb; 4SD, Four-Stair Down; 6MWT, Six-Minute Walk Test; 10MWRT, Ten-Meter Walk -Run Test.

In routine practice, clinicians rated the NSAA and 6MWT most favorably across all structural and qualitative domains, noting high relevance (92%-100%), quantifiability (89%-100%), feasibility (89%-100%), validity (87%-95%), and objectivity (78%-89%) (Table 3). The TTRF and PUL achieved intermediate ratings, with 60% to 90% of clinicians endorsing their relevance and feasibility. Conversely, the EK-2 scale received the lowest quality scores; among pediatric neurologists, only 67.6% deemed it relevant, 59.5% valid, and 48.6% objective. A significant operational readiness gap emerged regarding the technical psychometric properties of these functional scales across all disciplines. When asked to evaluate formal psychometric properties, specifically scale sensitivity to change and specificity, “no opinion” responses exceeded 45% among pediatric neurologists.

### 3.2 Perceived clinical value and willingness to adopt gene therapy

Despite limited prior clinical experience with gene therapy (reported by 35.1% of pediatric neurologist), overall receptivity was high, with 67.6% demonstrating strong willingness to adopt gene therapy in clinical practice (Table 4). Assessment of clinician familiarity with DMD gene therapy literature revealed that 43.2% of pediatric neurologists found published clinical trial evidence easily accessible and comprehensible, whereas 27.0% found it difficult to navigate (Table 4). Perceptions of clinical utility were positive: a substantial majority rated gene therapy as at least moderately effective in improving overall patient outcomes (78.4%) and slowing progressive muscle weakness (81.0%). Furthermore, virtually all surveyed clinicians anticipated meaningful, positive impacts on patient health-related quality of life. Consistent with this forward-looking perspective, 89.2% of participants agreed that introducing disease-modifying therapies provides significant clinical value to routine practice.

**Table 4:** Perceived utility, ease of use, and behavioral intentions of pediatric neurologists toward gene therapy for DMD (*n* = 37).

Prior clinical experience with gene therapy, n (%)		13 (35.1)
Perceived clinical effectiveness of gene therapy, n (%)	Not effective at all	0 (0.0)
	Slightly effective	8 (21.6)
	Moderately effective	19 (51.4)
	Very effective	9 (24.3)
	Extremely effective	1 (2.7)
Perceived utility in mitigating muscle weakness progression, n (%)	Not useful at all	0 (0.0)
	Slightly useful	7 (18.9)
	Moderately useful	17 (45.9)
	Very useful	11 (29.7)
	Extremely useful	2 (5.4)
Anticipated impact on patient health-related quality of life, n (%)	No impact	0 (0.0)
	Low impact	2 (5.4)
	Moderate impact	14 (37.8)
	High impact	17 (45.9)
	Very high impact	4 (10.8)
Perceived clinical value, n (%)	Strongly disagree	0 (0.0)
	Disagree	1 (2.7)
	Neutral	3 (8.1)
	Agree	21 (56.8)
	Strongly agree	12 (32.4)
Comprehensibility of clinical trial evidence, n (%)	Very difficult	0 (0.0)
	Somewhat difficult	10 (27.0)
	Neutral	11 (29.7)
	Somewhat easy	15 (40.5)
	Very easy	1 (2.7)
Clarity and accessibility of prescription guidelines, n (%)	Not clear at all	2 (5.4)
	Slightly clear	5 (13.5)
	Neutral	6 (16.2)
	Clear	13 (35.1)
	Very clear	5 (13.5)
	No prior experience	6 (16.2)
Anticipated workflow integration for therapy administration, n (%)	Strongly disagree	1 (2.7)
	Disagree	8 (21.6)
	Neutral	8 (21.6)
	Agree	18 (48.6)
	Strongly agree	2 (5.4)
Perceived logistical, multidisciplinary, and monitoring challenges, n (%)	Not challenging at all	1 (2.7)
	Slightly challenging	6 (16.2)
	Neutral	5 (13.5)
	Challenging	24 (64.9)
	Extremely challenging	1 (2.7)
Enthusiasm toward changing the current standard of care, n (%)	Not enthusiastic at all	0 (0.0)
	Slightly enthusiastic	2 (5.4)
	Neutral	2 (5.4)
	Enthusiastic	21 (56.8)
	Extremely enthusiastic	12 (32.4)
Perception that clinical benefits outweigh therapeutic risks, n (%)	Strongly disagree	0 (0.0)
	Disagree	1 (2.7)
	Neutral	9 (24.3)
	Agree	21 (56.8)
	Strongly agree	6 (16.2)
Willingness to recommend therapy to colleagues, n (%)	Not willing at all	1 (2.7)
	Slightly willing	2 (5.4)
	Neutral	4 (10.8)
	Willing	23 (62.2)
	Extremely willing	7 (18.9)
Openness to adopting gene therapy in routine practice, n (%)	Strongly disagree	0 (0.0)
	Disagree	1 (2.7)
	Neutral	2 (5.4)
	Agree	21 (56.8)
	Strongly agree	13 (35.1)
Likelihood of adoption if administrative burden is high, n (%)	Not likely at all	0 (0.0)
	Unlikely	0 (0.0)
	Neutral	3 (8.1)
	Likely	14 (37.8)
	Extremely likely	20 (54.1)
Likelihood of recommending to caregivers of early-stage patients, n (%)	Not likely at all	0 (0.0)
	Unlikely	1 (2.7)
	Neutral	2 (5.4)
	Likely	14 (37.8)
	Extremely likely	20 (54.1)
Likelihood of adoption if systemic economic impact is high, n (%)	Not likely at all	0 (0.0)
	Unlikely	0 (0.0)
	Neutral	2 (5.4)
	Likely	20 (54.1)
	Extremely likely	15 (40.5)

DMD, Duchenne Muscular Dystrophy.

### 3.3 Barriers, facilitators, and behavioral intentions regarding gene therapy implementation

Pediatric neurologists found clinical trial evidence and structural guidelines accessible but anticipated operational bottlenecks (Table 5). Over two-thirds (67.6%) characterized the complete delivery pathway, encompassing hospital logistics, multidisciplinary coordination, and mandatory long-term surveillance, as challenging or extremely challenging. Only 54.0% anticipated smooth workflow integration. Nonetheless, clinical enthusiasm remained robust: 89.2% felt enthusiastic or extremely enthusiastic about changing the standard of care. Benefit-risk perceptions were highly favorable, with 73.0% agreeing that clinical benefits outweigh potential risks, and 91.9% expressing high openness to adoption in personal practice.

**Table 5:** Perceived operational barriers and facilitators to gene therapy implementation among pediatric neurologists (*n* = 37).

Implementation barrier	Mean (SD)	Range (Min-Max)
Concerns regarding acute safety problems after infusion	2.30 (1.35)	1.0-6.0
Uncertainty regarding long-term efficacy	2.70 (1.66)	1.0-6.0
High cost and reimbursement constraints	3.38 (1.96)	1.0-7.0
High administrative or institutional burden	4.54 (1.83)	1.0-7.0
Absence of formal clinical guidelines or protocol frameworks	4.57 (1.72)	1.0-7.0
Potential resistance from patients or family caregivers	5.19 (1.76)	1.0-7.0
Operational and infrastructural burden of infusion	5.32 (1.38)	2.0-7.0

**Facilitating factor**	N (%)	
Availability of more robust clinical trial evidence	32 (86.5)	
Formal guidelines deployment from Scientific Societies	29 (78.4)	
Mitigation of institutional administrative workloads	22 (59.5)	
Provision of accessible, patient-friendly educational materials	19 (51.4)	
Direct positive recommendations from clinical colleagues	17 (45.9)	

DMD, Duchenne Muscular Dystrophy.

Most participants confirmed they were likely or extremely likely to adopt or recommend gene therapy even when facing high administrative burdens (91.9%) or significant economic impacts (94.6%). Based on mean relevance rankings (where lower scores indicate greater barrier relevance), the primary barriers identified by pediatric neurologists were acute post-infusion safety concerns (mean rank: 2.30), uncertainty regarding long-term efficacy (mean rank: 2.70), and high cost or reimbursement constraints (mean rank: 3.38). Conversely, key facilitators included robust clinical evidence (86.5%), professional society guidance (78.4%), and reduced administrative workloads (59.5%).

### 3.4 Predictors of willingness to adopt gene therapy

Multivariate logistic regression analysis identified technical confidence in specific functional assessments tools as the primary driver of behavioral intent. Pediatric neurologists with a high willingness to adopt gene therapy were significantly more likely to evaluate the 6MWT as an objective and sensitive functional instrument compared to those with lower willingness (96.0% vs. 41.7%; *p* = 0.0002). This perceived technical robustness of the 6MWT emerged as the sole independent predictor of high adoption intent in clinical practice (OR = 15.23, 95% CI: 1.58-146.67; *p* = 0.018).

In contrast, clinician experience profiles (including years managing DMD, neuromuscular specialization, or patient volume) did not significantly influence therapeutic readiness, as adoption willingness remained uniformly high across all sub-cohorts. Similarly, psychosocial and work-related behavioral scales revealed no statistically significant associations with adoption intent. Care-related regret, work engagement, and burnout failed to correlate with therapeutic readiness. Openness to innovation also did not statistically discriminate between high and low willingness cohorts, although descriptive trends showed that pediatricians with higher EBPAS scores perceived greater utility and quality-of-life impacts than their lower-scoring peers.

## 4 Discussion

This study characterizes baseline clinical readiness, awareness, and behavioral determinants regarding the integration of gene therapies for DMD. By evaluating primary stakeholders, this work addresses a critical translation gap between molecular innovation and health-system execution (13, 16, 19). From a public health perspective, our findings demonstrate that high clinician willingness to adopt gene therapy coexists with significant gaps in institutional infrastructure, operational readiness, and technical familiarity with functional assessment tools.

The management of DMD is undergoing a structural transition from palliative care toward genotype-specific, disease-modifying interventions (11, 13, 37). While multidisciplinary care and corticosteroids historically served as the sole natural-history-modifying strategies, newer agents like vamorolone address chronic muscle inflammation without correcting the underlying genetic defect (38, 39). Recombinant adeno-associated virus vector gene therapies represent a major milestone in targeting this root cause (1, 10, 11, 19, 20). In Europe, current regulatory latency surrounding delandistrogene moxeparvovec creates a unique window for proactive planning (24). Indeed, 67.6% of surveyed pediatric neurologists demonstrated high baseline willingness to adopt gene therapy despite lacking real-world exposure, an initial receptivity that health authorities can leverage during infrastructure development.

An important finding of this study was the association between pediatric neurologistś perception of the 6MWT as an objective, sensitive instrument and their willingness to adopt gene therapy (OR = 15.23). We hypothesize that this stems from the continuous, highly quantifiable nature of the 6MWT (distance walked in meters), which provides clinicians with the diagnostic certainty needed to baseline patients, track post-infusion functional trajectories, and objectively justify treatment initiation when deploying high-cost, high-risk biologicals. Thus, confidence in standardized outcome measures lowers perceived barriers to integrating novel therapeutics (1, 10). However, over 45% of surveyed pediatric neurologists selected “no opinion” regarding the formal sensitivity to change and specificity of standard functional scales, highlighting a critical clinical readiness gap (3, 13, 37, 40, 41). Although the 6MWT is a standard regulatory endpoint, over-reliance on a single metric overlooks inherent vulnerabilities, including effort dependence and non-linear disease trajectories (1, 3, 10, 40). This limitation, coupled with a widespread lack of psychometric familiarity, poses significant challenges for real-world effectiveness tracking and value-based reimbursement frameworks, underscoring the urgent need for health authorities to mandate multi-dimensional assessment guidelines and targeted interprofessional education prior to broader deployment.

While clinical enthusiasm is high, long-term therapeutic durability and administration timing present major operational challenges (9, 20, 37). Although 5-year trial outcomes for delandistrogene moxeparvovec show sustained functional divergence from natural history, the non-integrating nature of episomal recombinant adeno-associated virus vectors raises concerns regarding potential transgene dilution during pediatric muscle growth (22, 42). Conversely, delaying treatment in older patients risks delivering vector genomes to irreversible, fibrotic muscle tissue.

Safety considerations emerged as the primary implementation barrier. High-dose systemic administration of adeno-associated virus vectors genomes can trigger life-threatening acute toxicities, including immune-mediated hepatotoxicity, myocarditis, thrombotic microangiopathy, and treatment-related mortality, alongside subacute transgene-directed T-cell responses (14, 43, 44). These severe risks justify strict institutional preparedness criteria (3, 13, 44). Safe administration requires specialized tertiary neuromuscular reference centers equipped with pediatric intensive care capacity, immediate access to subspecialists (cardiology, hepatology, immunology), and standardized protocols for real-time safety surveillance and acute triage (12, 13, 17, 45).

Historically, limited treatment options restricted the scope of shared decision-making in DMD care (1, 41, 44). As therapeutic options expand, healthcare systems must support shared decision-making using plain-language tools and decision aids tailored to family health literacy (1, 9, 13). Discussions must systematically balance potential clinical benefits against therapeutic uncertainties and treatment-related burdens (10, 46, 47). Executing this complex care model relies on a coordinated interprofessional approach, including pediatric neurologists, cardiologists, genetic counselors, rehabilitation specialists, nurses, and social workers (10, 13). Within this framework, hospital pharmacists provide essential stewardship over cold-chain logistics, weight-based compounding, vector shedding precautions, and toxicological monitoring (48, 49).

This study has several methodological limitations. First, because this study evaluates a pre-approval setting where most participants lack direct clinical experience with DMD gene therapy, the survey captures self-reported perceptions and hypothetical intent rather than audited clinical practice. Stated willingness in hypothetical scenarios does not consistently predict actual behavior and can either overestimate or underestimate participation once real-world administrative and operational burdens materialize (50). Consequently, these findings reflect baseline professional attitudes and health-system bottlenecks rather than definitive post-authorization prescribing patterns. Second, recruitment through national scientific societies yielded a sample enriched with tertiary academic clinicians, which may reflect an upper tier of institutional preparedness rather than a uniform national baseline. Third, the modest sample size constrained statistical power for highly stratified sub-analyses across stakeholder cohorts. Fourth, while our survey assessed general trial evidence comprehensibility and overall benefit-risk perceptions, it did not evaluate which specific clinical endpoints (e.g., delay in loss of ambulation, respiratory preservation) or evidential thresholds clinicians find most persuasive. Because clinicians routinely leverage their professional expertise to assess the desirability and evidential basis of novel interventions, future implementation studies should investigate these specific expectations to clarify the clinical benchmarks required to translate high theoretical receptivity into confident real-world adoption. Finally, while our study captures the perspectives of primary prescribers and core allied health professionals, it did not include other key multidisciplinary team members, such as genetic counselors, nurse coordinators, or clinical geneticists, who play critical roles in pre-treatment genetic screening, family counseling, and long-term care coordination. Furthermore, structural determinants of technology adoption, such as hospital budget allocation, institutional priority setting, resource distribution, and executive leadership support for cross-departmental workflow restructuring, remain outside the direct control of frontline clinical practitioners. Consequently, managerial, organizational, and health-system leadership factors, along with broader multidisciplinary stakeholder cohorts, were not directly evaluated, representing an essential domain for future health-policy research.

## 5 Conclusions

This study demonstrates a highly receptive yet operationally constrained clinical landscape for adopting DMD gene therapies. Although specialized pediatric neurologists exhibit strong behavioral intentions to implement these advanced therapeutics, safe clinical translation remains hindered by technical gaps in functional scale mastery, limited clinical infrastructure, and severe post-infusion safety risks. Optimizing care delivery requires standardized interprofessional monitoring protocols, targeted cross-disciplinary psychometric education, and systematic real-world safety surveillance. To navigate this evolving therapeutic paradigm, healthcare authorities, hospital administrators, and executive leadership must invest in institutional preparedness while aligning clinical readiness with innovative risk-sharing, manufacturing, and managed-access procurement models to safeguard public healthcare budgets and ensure sustainable, equitable patient access.

## Data Availability

Researchers may request access to individual patient level data through the clinical study data request platform (vivli.org). Further details on Roche's criteria for eligible studies are available at vivli.org/ourmember/roche. For further details on Roche's Global Policy on the Sharing of Clinical Information and how to request access to related clinical study documents, see www.roche.com/innovation/clinical-trials/data-sharing.
